# Junior doctors rate online simulation as ‘good enough’ but not as good as face to face sessions

**DOI:** 10.1192/bjo.2021.75

**Published:** 2021-06-18

**Authors:** Josh Bachra, Anna Ludvigsen, Kehinde Junaid

**Affiliations:** Nottinghamshire Healthcare NHS Foundation Trust

## Abstract

**Aims:**

To compare the feasibility and acceptability of delivering a simulation-based learning (SBL) programme for Junior Doctors virtually versus face to face.

**Method:**

The Nottinghamshire Healthcare Simulation Centre has been delivering a SBL programme for Foundation Year 2 doctors on behalf of Health Education East Midlands for the past three years. Since face to face teaching was not possible during the COVID-19 pandemic the programme was delivered online using the same content and format as for prior cohorts. Feedback questionnaires from 128 face to face participants (F2F) and 133 virtual participants (V) were compared.

**Result:**

There was a decrease in Likert scale ratings across all domains in the virtual group. This was most apparent when examining the ‘strongly agreed’ responses: the venue/remote format was suitable for the session 34% decrease, the course length was appropriate 24% decrease, the pace of the course was appropriate 20% decrease, the simulation was helpful and relevant 15% decrease, the content of the course was organised and easy to follow 13% decrease, the learning objectives were met 10% decrease, the presenters were engaging 6% decrease, the trainers were well prepared 3% decrease. The virtual group included responses in the ‘strongly disagree’ and ‘disagree’ categories relating to the virtual format, length and pace, which did not occur in any domain for the F2F group.

Combining the ‘strongly agree’ and ‘agree’ statements also showed a decrease in satisfaction with 72.5% of responses falling into this category for the V group and 88.3% for the F2F group. Fewer participants in the V group would recommend the course to a colleague (98% V vs 99% F2F).

**Conclusion:**

Providing the SBL programme using an online format was feasible while also being acceptable to most participants. However, participants did not rate this experience as highly as face to face teaching. The largest decreases in satisfaction were in areas related to the virtual format. An interesting finding is that participants rated the pace and length of the online course as less agreeable, despite the content and scheduling being the same as for the face to face group.

Based on these findings face to face teaching should resume when practicable. In the meantime, the virtual delivery may be improved if the course length was reduced. Analysis of qualitative feedback may provide insights into why participants did not rate the virtual simulation as highly as the face to face equivalent.

